# The lumbar spinal endplate lesions grades and association with lumbar disc disorders, and lumbar bone mineral density in a middle-young general Chinese population

**DOI:** 10.1186/s12891-023-06379-w

**Published:** 2023-04-03

**Authors:** Jingzhe Pei, Aihong Yu, Jian Geng, Yandong Liu, Ling Wang, Jia Shi, Fengyun Zhou, Tianyu Zhang, Pengju Huang, Xiaoguang Cheng

**Affiliations:** 1 Department of Radiology, Beijing Jishuitan Hospital, 31 Xinjiekou East Street, Xicheng District, Beijing, China; 2 Shaanxi University of Chinese Medicine School of Medical Technology, Middle Section of Shiji Avenue, Xixian New District, Xianyang City, Shaanxi Province China; 3 Chinese Center for Disease Control and Prevention, National Institute of Nutrition and Health, 29 Nanwei Road, Xicheng District, Beijing, China

**Keywords:** Endplate lesions, Lumbar intervertebral disc herniation, Pfirrmann grading, Volumetric bone mineral density

## Abstract

**Background:**

Lumbar vertebral endplates lesions (LEPLs), one of the etiologies of low back pain (LBP), are one of the most prevalent causes of health-care costs. Despite progressively becoming the focus in recent years, almost all studies have concentrated on symptomatic patients rather than general populations. As a result, our study was designed to determine the prevalence and distribution patterns of LEPLs in a middle-young general population, as well as their associations with lumbar disc herniation (LDH), lumbar disc degeneration (LDD), and lumbar vertebral volumetric bone mineral density (vBMD).

**Methods:**

Seven hundred fifty-four participants aged 20–60 years were recruited from the subjects enrolled in a 10-year longitudinal study of degeneration of the spine and knee being conducted at the Beijing Jishuitan Hospital and 4 of them were excluded due to the missing of MRIs. In this observational study, a lumbar quantitative computed tomography (QCT) and MRI scan were performed among participants within 48 h. T2-weighted sagittal lumbar MRI images for all included subjects were identified for LEPLs by two independent observers based on morphological and local characteristics. Lumbar vertebral vBMD was measured with QCT. The age, BMI, waistline, hipline, lumbar vBMD, LDD, and LDH were measured to investigate their associations with LEPLs.

**Results:**

The prevalence of LEPLs was higher among the male subjects. 80% of endplates were recognition as no lesions with a substantial disparity between female (75.6%) and male subjects (83.4%) (*p* < 0.001). The most common lesions were “wavy/irregular” and “notched”, and “fracture” is most involved in L3-4 inferior endplate both in two genders. LEPLs were found to be associated with LDH (≥ 2 levels: OR = 6.859, *P* < 0.001; 1 level: OR = 2.328, *P* = 0.002 in men. OR = 5.004, *P* < 0.001; OR = 1.805, *P* = 0.014 in women) reference for non-LDH, and hipline in men (OR = 1.123, *P* < 0.001).

**Conclusions:**

LEPLs are the common findings on lumbar MRIs in general population, particularly in men. The presence of these lesions and advance from slightly to severely could be mainly attributed to LDH and men’s higher hipline.

## Introduction

Low back pain (LBP), which presents the world’s highest burden of disease related to years lived with disability in both developed and developing countries, is a multifactorial symptom, while standard diagnostic approaches usually fail to reveal any pathoanatomical cause of the pain within the cognition of modern medicine [1, 2]. Lumbar vertebral endplates lesions (LEPLs), as one of the etiology of LBP [3], have been gradually regarded as the focus in recent years [4–6]. The most common endplate lesions observed is probably the Schmorl’s node, which is regarded as a herniation of nucleus pulposus through the focal weak spots of cartilaginous and bony endplate into the body of the adjacent vertebrae. Schmorl’s nodes begin as small defects and usually are viewed as synonymous with LEPLs, especially in radiological studies. Chen et al. revealed that it was endplate defects, that represented the dominant association with lifetime back pain [3].

The perception of LEPLs' pathogenesis is ambiguous, and there is little knowledge about how LEPLs connect to lumbar disc diseases like lumbar intervertebral disc herniation (LDH) and lumbar disc degeneration (LDD). The endplats (EPs), which are the connective tissue between the vertebrae and the intervertebral disc, are crucial for keeping the nutritional supply, the molecular communication and health of the disc and absorbing pressure from mechanical loading from spinal activities [7]. The modifications in the biomechanical and biochemical properties of EPs are associated with LBP development. Since the disc is an avascular structure, nutrients from the circulation must diffuse across the EPs and disc matrix before reaching the disc cells. Contrary to the discs, the central endplate and the nearby vertebral marrow are both well innervated, providing a solid physiological foundation for the central endplate to serve as the main pain generator. Additionally, most of their studies were concentrated on patients with chronic low back pain, despite the fact that recent studies have harmonized the LEPLs and their nomenclature scheme and reduced inconsistency by adopting a universal nomenclature [4, 8].

Although the presents of endplates lesions and lumbar disc disorders frequently occur in general people without LBP, the prevalence of LEPLs in these individuals has received little research. Furthermore, the association of endplates lesions with the lumbar vertebral trabecular volumetric bone mineral density (Trab.vBMD, hereinafter referred to as the “vBMD”) is unclear [9–11]. The objective of the present study is to investigate the prevalence of LEPLs in affected levels (L1/L2–L5/S1) and detect possible associations of the presence and advance of the LEPLs with potential risk factors, including characteristics data, lumbar vBMD and the grade of LDD and LDH in a middle-young Chinese cohort.

## Methods

### Study design and population

The subjects were from a 10-year longitudinal study on the degeneration of the spine and knee performed running in June 2014, and the basic data collection of this population was completed in 2017. Participants who are healthy community-dwelling adults, aged 20–60 years, not engaged in heavy physical activity, and residents in Beijing > 5 years were included to facilitate follow-up and reveal the onset and development of the degeneration. Therefore, we use the baseline data to design a cross-sectional study to investigate LEPLs. The study was approved by the ethics committee of our hospital. The study was conducted under the Helsinki Declaration. Informed consent was obtained from all individual participants included in the study. The exclusion criteria were: autoimmune disease (e.g., rheumatoid arthritis), congenital disorders (e.g., juvenile idiopathic scoliosis), prior lumbar spine surgery, a history of metabolic bone disease, or chronic diseases related to calcium absorption (hyperparathyroidism), a history of malignant tumors, the use of medications known to affect bone metabolism, and pregnancy [12].

After enrolling, 4 of the 754 subjects were removed from the study since their lumbar MRIs were unavaliable. In our study, therefore, 750 middle-young participants who had experienced a lumbar quantitative computed tomography (QCT) and MRI scan within 48 h were analyzed. The lumbar spine disorders such as LDH, LDD (decreased signal intensity and losing the height of disc), and LEPLs were assessed by the radiologists by MRI examination performed by a 1.5 T scanner (Espree, SIEMENS, Munich, Germany). All subjects were scanned with the same multi-channel gradient waist coil. T2-weighted TSE imaging (TR/TE 2500/100 ms, slice thickness 4 mm, intersection gap 0.8 mm, voxel 240 × 282, FOV 180 × 280 mm) was performed in the sagittal plane. In the axial plane, T2-weighted TSE imaging was performed with TR/TE 2500/100 ms, slice thickness 4 mm, intersection gap 0.4 mm, voxel 248 × 198, and FOV 160 × 180 mm.

As part of the study protocol, the lumbar spines of all-volunteer scans were performed on a Toshiba CT scanner (Aquilion PRIME, Toshiba, Otawara, Japan). A QCT calibration phantom (Mindways Inc., Austin, TX, USA) was placed beneath the spine and scanned simultaneously according to the standard scanning protocol by Wang et al. [13]. The spine was kept parallel to the long axis of the calibration phantom, and minimal air gaps existed between the phantom and the volunteer. The scanning parameters were as follows: 120 kV 187 mAs, the field of view 50 cm, 1 mm slice thickness, and reconstruction matrix 512 × 512. Other methodological details have been described previously [14].

Demographic (sex, age), and physical (weight, height, waistline, and hipline) characteristics were collected from the enrolled population. Body mass index (BMI) was acquired as weight (kg) / height squared (m^2^).

### Scoring system for the LEPLs, LDD and LDH

Two independent radiologists analyzed the T2-weighted sagittal and axial images under the guidance of a senior radiologist in order to identify the LEPLs, LDD, and LDH. They did so while remaining blind to one another to avoid detection bias.

**Fig. 1:**
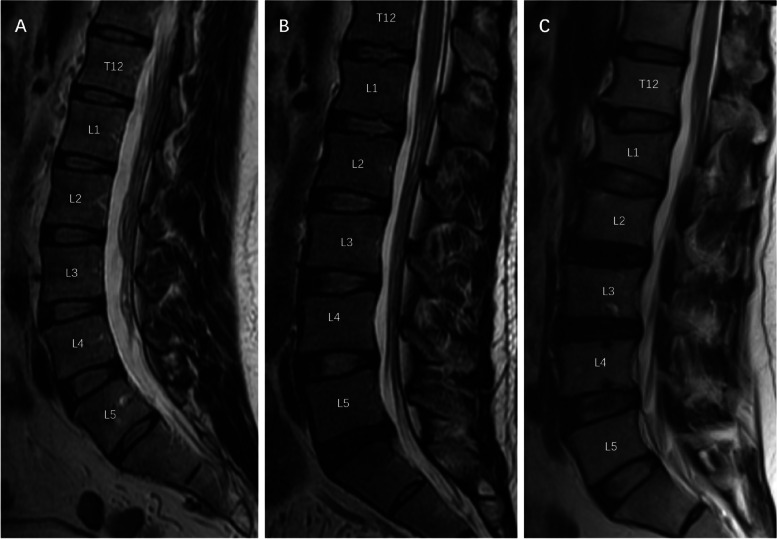
Examples of lumbar vertebral endplates lesions (LEPLs) on T2-weighted sagittal MRI scans in three grades. **A** A 33-year-old male with smooth ten lumbar endplates instead of LEPLs, was assigned to the non-LEPLs group and graded to Grade I. **B** A 40-year-old male with four notched endplates involving L1-2 and L2-3 disc both cranial and caudal endplates and with the notched occur in L3-4 caudal endplate, was designated as mild-LEPLs and graded to Grade III. **C** A 51-year-old female, with a sagittal MR image of a lumbar spine with two Schmorl’s nodes which were observed at the caudal endplate of the L3-4 disc and the cranial endplate of the L4-5 disc, two fracture endplates were found at the caudal endplate of the T12-L1 and L2-3 disc, besides two wavy endplates were found at the caudal endplate of the L1-2 and L2-3 disc, was evaluated as severe-LEPLs and graded as Grade III

Ten endplates in the lumbar spine (L1-S1) were evaluated for the presence or absence of any type of defect. The score was calculated by labeling L1/L2 to L5/S1 intervertebral spaces as: “normal (1 point) = no lesions in the intervertebral space, physiological curvature of both EPs; wavy/irregular (2 points) = no specific lesions detectable in the intervertebral space, alterations in the physiological curvature of at least one of the EPs; notched (3 points) = a V-shaped or circular small lesion visible in at least one sagittal MRI slice; Schmorl’s node (4 points) = a deep focal defect of the vertebral EP with a smooth margin and a rounded appearance; fracture (5 points)” as previously reported by Brayda-Bruno et al. [4, 6]. For each of the five intervertebral levels, if two or more defects co-existed on an endplate or there were two types of defects in a disc level (including the upper and lower endplates), the higher score was counted. To conduct a comprehensive score for LEPLs of each individual, we define non-LEPLs (Grade I) when the upper and lower endplates of each of the five lumbar intervertebral discs were normal, mild LEPLs (Grade II) when one or more of the EPs was scored as 2(wavy/irregular) or 3(a V-shaped or circular small lesion) points, and severe LEPLs (Grade III) when the presentations of 4/5 points were evaluated (Fig. 1).

MRI degeneration of the L1/2–L5/S1 intervertebral discs in the whole cohort were assessed using a 5-point scoring system proposed by Pfirrmann et al. [15] as follows: Grade I: the homogeneous disc is with a bright hyperintense white signal intensity and a normal disc height. Grade II: The inhomogeneous disc with a hyperintense white signal and a normal disc height, and with or without horizontal gray bands. Grade III: the inhomogeneous disc with an intermediate gray signal intensity, with a normal or slightly decreased height, and indistinction between nucleus and annulus. Grade IV: the inhomogeneous disc with a hypointense dark gray signal intensity, with a normal or moderately decreased disc height, and the distinction between nucleus and annulus is lost. Grade V: the inhomogeneous disc with hypointense black signal intensity, the distinction between the nucleus and annulus is lost, and its space is collapsed. The intervertebral discs (IVD) were then classified into 3 groups. A subject was defined as having slight degeneration if their average grade of Pfirrmann grade was < III, moderate degeneration if their average grade was ≥ III and < IV, and severe degeneration if their grade was ≥ IV [16].

To describe the overall lumbar intervertebral disc herniation of each subject, we defined the non-LHD group as follows: normal, or bulge/protrusion with no evident contact of disk material with the nerve root, and without severe thecal sac compression or diminished dimensions of the neural foramen. The LDH group was defined as follows: the bulge, protrusion with the visible contract of disk material with the nerve root, severe thecal sac compression, and diminished dimensions of the neural foramen, extraction, and sequestration. The number of discs with herniation counts is distributed into the following 3 groups, non-lumbar intervertebral disc herniation, a single-level disc herniation, and ≥ 2 segments herniation.

### Lumbar Vertebral Trabecular Volumetric Bone Mineral Density (Trab.vBMD) Measurement

After scanning, the CT DICOM images were transferred to the QCT workstation for further analysis with the QCT Pro 5.0.3 software (Mindways Inc.). lumbar vBMD was measured within a specific region of interest, which was defined as the oval-shaped areas containing the largest areas of the trabecular bone in the mid-plane of each vertebral body, not including the cortical bone or basivertebral vein [14, 17]. The Trab.vBMD values (mg/cm3) of L2–4 were recorded and analyzed, respectively, and the average was calculated [17].

### Statistical analysis

All 30 out of 750 MRIs that were being re-evaluated by were used to assess inter- and intra-observer reliability of involveing radiologists in the discrimination of LEPLs, LDD and LDH. Strong consistency is demonstrated by the Kappa-values in distinguishing LEPLs, LDD, and LDH, which are 0.87, 0.91, and 0.94 for the intra-observer and 0.66, 0.79, and 0.88 for the inter-observer, respectively.

The data were stratified into the male and female groups and then the normality was tested in the continuous variables. The descriptive statistics were presented as mean ± standard deviation for the normally distributed variables and as a median for the categorical and non-normally distributed variables. Then, the data were stratified by lumbar endplates condition, the normally distributed variables were analyzed using the one-way analysis of variance (ANOVA), and others were evaluated with the Kruskal–Wallis H test. The linear relationship of LEPLs with the stage of LDH and LDD was analyzed by Mantel–Haenszel chi-square tests. Finally, the ordinal logistic regression(OLR) was performed to estimate the effect of age, BMI, waistline, hipline, lumbar vBMD, lumbar intervertebral disc herniation, and lumbar disc degeneration on lumbar endplates lesions status and adjusting for confounding factors. Odds ratios (ORs) and 95% confidence intervals (CIs) for the occurrence of LEPLs were calculated as approximations of the relative risk estimates. A *P*-value < 0.05 was considered statistically significant. SPSS 26.0 software was used to perform statistical analysis.

## Results

### Demographic characteristics

Seven hundred and fifty participants (326 men, median age 39 years old; 424 women, median age 40 years old) were enrolled in the study for analysis. Since the characteristics, mean lumbar vBMD, and the prevalences of LDH, LDD and LEPLs of this cohort are incomparable between both genders, they are dichotomous in the analyses and exhibitions. These characteristics are shown in Table 1.

**Table 1:** Characteristics of subjects with different stages of the lumbar vertebral endplates lesions

	Female, *N* = 424				Male, *N* = 326			
Parameters	Non-LEPLs	LEPLs *n* = 251		P	Non-LEPLs	LEPLs *n* = 237		P
		Mild-LEPLs	Severe-LEPLs			Mild-LEPLs	Severe-LEPLs	
%(n)	41.0(174)	42.0(178)	17.0(72)		27.3(89)	54.9(179)	17.8(58)	
Age(years)	38	40	45	< .001^*^	37	39	43.5	< .001^*^
Height (cm)	160.2 ± 5.4	163.1 ± 5.6	161.0 ± 5.9	.469^**^	170.7 ± 6.2	172.6 ± 5.9	173.6 ± 5.2	.008^**^
Weight (Kg)	59	60	61	.21^*^	75	77	80.5	.013^*^
BMI	24.7	23.2	23.5	.556^*^	25.9	25.5	27.0	.275^*^
Waistline(cm)	78	79	79	.638^*^	90	90	92	.155^*^
Hipline(cm)	95	95	97	.314^*^	98	101	101.8	< .001^*^
Mean vBMD(mg/cm^3^)	168.4 ± 35.1	163.1 ± 38.1	156.1 ± 33.5	0.049^**^	153.5 ± 30.3	149.7 ± 29.7	135.5 ± 34.4	0.002^**^
The status of LHD								
Non-LHD n (expected n)	62 (44.1)^a^	38 (45.3)	8 (18.6)^a^	< .001^***^	41 (23.5)^a^	38 (47.2)	7 (15.3)	< .001^***^
1-level LHD n (expected n)	84 (82.0)	94 (84.4)	23 (34.6)		40 (43.7)	100 (87.97)	20 (28.5)	
≥ 2 levels LHD n (expected n)	27 (46.9)^a^	446 (48.3)	42 (19.8)^a^		8 (21.8)^a^	41 (43.9)	31 (14.2)^a^	
The LDD stages								
Slight n (expected n)	78 (69.0)	74 (70.9)	17 (29.1)^a^	< .001^***^	48 (38.2)	78 (76.9)	14 (24.9)^a^	< .001^***^
Moderate n (expected n)	93 (98.7)	99 (101.6)	50 (41.7)		41 (49.7)	99 (99.9)	42 (32.4)	
Severe% n (expected n)	2(5.3)	5(5.5)	6(2.2)		0 (1.1)	2 (2.2)	2 (0.7)	

*LEPLs* lumbar endplates lesions (EPLs), *Non-LEPLs* the score of the LEPLs is 1 point, *mild-LEPLs* the score of the LEPLs is 2/3, and severe-LEPLs: the score of the LEPLs is 4/5; 1 to 5 points represent normal, wavy/irregular, notched, Schmorl’s node, and fracture; *BMI* Body mass index, *vBMD* Volumetric bone mineral density, *LDH* Lumbar intervertebral disc herniation, *LDD* Lumbar intervertebral disc degeneration

^*^by Kruskal–Wallis H test

^**^by one-way variance analysis (ANOVA)

^***^by Mantel–Haenszel chi-square tests

^a^absolute value of adjusted standardized residual is greater than 3

### Prevalence and distribution of lumbar endplates lesions

**Fig. 2:**
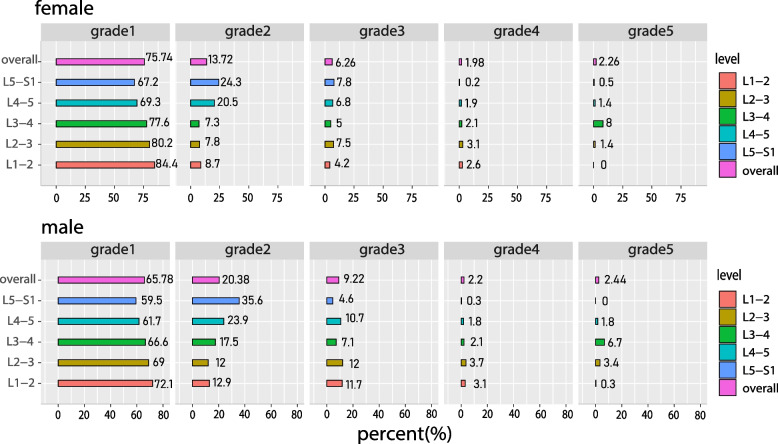
The publication of the endplates lesions in each lumbar intervertebral level both in women and men. Grade 1 represents “normal”, grade 2 “wavy/irregular”, grade 3 “notched”, grade 4 “Schmorl’s node”, and grade 5 surrogates fracture

LEPLs were identified in 1500/7500 (20.0%) endplates, 1072/3750 (28.6%) levels of discs, and 487/750 (64.9%) subjects. Grades II and III of LEPLs were assigned to 178 (42.0%) and 73 (17.2%) women and 179 (54.9%) and 58 (17.8%) males, respectively (shown in Fig. 2). The predilection for men over women in LEPLs (72.7% vs. 59.0%, *p* < 0.001), and the positive rates of both sexes increased with the descent of the lumbar segment (from 15.6% to 32.8% in women and from 29.7% to 40.5% in men) (shown in Table. 2). The type of LEPLs most commonly observed in both the sex was the “wavy/irregular”, subsequently followed by the “notched”, “fracture” and “Schmorl’s node”.

**Table 2:** Incidence of lumbar vertebral endplates lesions in all participants

Parameters	Total		Female		Male		P
	n	%	n	%	n	%	
Normal^a^	263	35.1	174	41.0	89	27.3	< 0.001
1-level	192	25.6	111	36.2	81	34.8	0.6785
2-levels	116	15.5	66	15.6	57	17.5	0.4818
3-levels	86	11.5	36	8.5	50	15.3	0.0035
4-levels	54	7.2	22	5.2	32	9.8	0.0151
5-levels	32	4.3	15	3.5	17	5.2	0.2600
Total	750	100	424	100	326	100	
Normal^b^	6000	80.0	3536	83.4	2464	75.6	< 0.001
Superior	761	10.1	356	8.4	405	12.4	< 0.001
Inferior	739	9.9	348	8.2	391	12.0	< 0.001
Total	7500	100	4240	100	3260	100	

^a^Percentage (%) to the total number of subjects is encompassed

^b^Percentage (%) to the total number of endplates is encompassed

Men and women have slightly different LEPLs distributions across the segment of five lumbar discs. In women, “wavy/irregular” and “notched” most frequently affected the L5-S1 segment, “Schmorl’s node” L2-3 segment, and “fracture” L3-4 segment, while in male, the distribution of “notched” and “Schmorl’s node” most commonly affected L2-3 segment. LEPLs slightly more affected the cranial endplates than the caudal endplates of lumbar spine (761 vs. 739) with the L4-5 level was a clear demarcation. Interestingly, endplates’ fractures” occur most frequently at the inferior endplate of L3-4 disc both in men and women.

### The association of LEPLs with LDH and LDD

Table 1 shows that while BMI did not change significantly between the three groups, participants with a greater degree of LEPLs were on average older (*P* < 0.001), had a lower average Trab.vBMD either males (*P* = 0.002) or females (*P* = 0.049), and were taller and heavier in males (*P* = 0.008 in height and 0.013 in weight). The status of LDH and LDD that were stratified by gender was also shown in Table 1. According to the Chi-square tests of linear-by-linear association, both in women and men, LEPLs had a linear relationship with LDH and LDD (the 4 *P* values were all less than 0.001). The results of post hoc tests between LEPLs and LDH implie that, in both men and women, the grade of LDH lesions increases with the increase of LEPLs grades, while the results between LEPLs and LDD suggest that individuals without severe LDD were not more likely to acquire a higher grades of LEPLs.

### Risk factors for the advancement of LEPLs

The OLR analysis for these factors is shown in Tables 3 and 4. The results show that among participants with 1 level LDH and 2 levels LDH, respectively, in men, the OR was 2.328 (*P* = 0.002) and 6.859 (*P* < 0.001), and in women, it was 1,805 (*P* = 0.014) and 5.004 (*P* < 0.001). The hipline of males was another factor that had a substantial impact on the progression of LEPLs (OR = 1.123, *P* 0.001). Although both lumbar vBMD and LDD were related to LEPLs, vBMD (OR = 0.997, *P* = 0.537 for men, and 1003, 0.55 for women) and LDD (OR = 2.501, *P* = 0.400 and OR = 1.313, *P* = 0.361, respectively in severely and moderately LDD in men,and OR = 1.539, *P* = 0.479 and OR = 0.818, *P* = 0.416 in women) reference for slightly LDD did not affect the progression of LEPLs after the calibration of covariates.

**Table 3:** The results of the ordinal logistic regression for lumbar verterbral endplates lesions in women

Parameter		Coefficient (B)	Wald Chi-Square	P	OR	95% Wald Confidence Interval for OR	
						Lower	Upper
Threshold of EPLs grade	Grade II	2.559	1.855	.173	12.924	.325	513.652
	Grade III	4.712	6.220	.013	111.236	2.743	4511.656
age		.028	3.490	.062	1.029	.999	1.060
BMI		-.045	.950	.330	.956	.874	1.046
waistline		-.009	.464	.496	.991	.964	1.018
hipline		.029	1.339	.247	1.029	.980	1.081
mean L BMD		.002	.358	.550	1.002	.996	1.008
The number of levels of LDH							
≥ 2 levels		1.610	27.557	.000	5.004	2.743	9.128
1 level		.591	6.069	.014	1.805	1.128	2.888
0 level		Reference	-	-	1	-	-
The grade of LDD							
severely		.431	.502	.479	1.539	.467	5.078
moderately		-.201	.662	.416	.818	.504	1.327
slightly		Reference	-	-	1	-	-

**Table 4:** The results of the ordinal logistic regression for lumbar verterbral endplates lesions in men

Parameter		Coefficient (B)	Wald Chi-Square	P	OR	95% Wald Confidence Interval for OR	
						Lower	Upper
Threshold of EPLs grade	Grade II	6.103	6.339	.012	447.122	3.865	51,730.770
	Grade III	9.047	13.552	.000	8494.497	68.746	1,049,603.691
age		.005	.066	.798	1.005	.969	1.042
BMI		-.081	1.554	.213	.922	.812	1.048
waistline		-.035	1.720	.190	.966	.916	1.017
hipline		.116	13.491	.000	1.123	1.055	1.194
mean L BMD		-.003	.381	.537	.997	.989	1.006
The number of levels of LDH							
≥ 2 levels		1.926	24.933	.000	6.859	3.221	14.607
1 level		.845	9.304	.002	2.328	1.353	4.006
0 level		Reference	-	-	1	-	-
The grade of LDD							
severely		.917	.708	.400	2.501	.296	21.137
moderately		.272	.834	.361	1.313	.732	2.356
slightly		Reference	-	-	1	-	-

## Discussion

Based on a T2-weighted MRI Classification system for the LEPLs and a lumbar spine QCT scanning for vBMD measurements, the associations between LEPLs and LDH, LDD, and lumbar vBMD were investigated in a general middle-young Chinese population. LEPLs were common findings in the male lumbar spine, particularly in the lower lumbar region. The presence of endplate lesions was associated with several factors, but these associations only remained with LDH and male hipline after adjusting for the effects of age, BMI, waistline, lumbar vBMD, and disc degeneration.

Our study showed remarkable gender differences not only in characteristics data and lumbar vBMD but also in the prevalence of lumbar disc disorders. Male gender is associated with a higher positive incidence for LEPLs than females whether from the prevalence of LEPLs and the number of the lesioned endplates, in which men often do harder physical labor than women [18], which might explain for a larger hipline accompanies and exacerbates the advance of LEPLs. Hormonal factors may play some as yet consequential role in the development of LEPLs [6]. Moreover, our study finds that except for one level, the incidence of LEPLs was lower in males than in females, and the incidence of other 2–5 levels was higher in males than in females.

Our results showed that LEPLs involved 263 (64.9%) general people, 1072 (28.6%) discs, and 1500 (20.0%) lumbar endplates, and this is corresponding with the previous study [3, 4, 8]. However, Wang et al. observed 1148 vertebral endplates (L1–S1) from the cadaveric spines of 136 men and found 45.6% of lumbar vertebral endplates with lesions [19]. Although, it is unexceptional that the prevalence of LEPLs is higher in men than women and even the whole cohort, the difference can be accounted for that the medical imaging is not on par with the pathological examination, and the two methods should not compare immediately without segregation in clinical studies and diagnoses.

Brayda-Bruno [4] et al. researched that “notched” was the most common type of LEPLs followed by “Schmorl’s node” observed in patients with low back pain, our studies reveal that the most common EPLs are “Wavy/irregular” and “notched”. This difference could account for our subjective population being the general people without low back pain conditions occur that can be severe enough to affect normal work and activity. Based on the fact that “Wavy/irregular” & “notched” are more common in general people, and “notched” & “Schmorl’s node” are more common in a person with low back pain, we can preliminarily infer that “Wavy/irregular” is physiological, while “notched” & “Schmorl’s node”, especially the latter, are more likely to be symptomatic or even pathological. At least, "Notched" and "Schmorl's Nodes" are risk factors for low back pain. This is also consistent with Chen’s finding that focal defects were statistically significantly associated with the presence of back pain over the past 12 months (OR = 2.10, *P* = 0.009) [3]. Moreover, we find that the fracture is most involved in L3-4 inferior endplate. This may be because the stress distinctiveness of L3-4 intervertebral discs at the boundary between the upper and lower lumbar spine is predisposed to mechanical failure.

Proteoglycan molecules are critical for the control of solute transport and maintenance of water content in the disc, and consumption of proteoglycans from the endplate cartilage is associated with loss of proteoglycans from the nucleus [7]. Then, a finite element analysis concludes that variations in proteoglycan content of the disc were associated with variations in high tensile strains in the endplates, which is responsible for vertebral compressive strength [20]. As a result, we found that LDH and LDD are associated with LEPLs, and LDH is one of the risk factors for LEPLs progression and is following previous studies [4, 8, 21, 22]. Moreover, Sahoo has identified that endplate lesions are commonly associated with symptomatic LDH, and bony lesions are affiliated with its adverse outcomes [23].

This is the first study determining the role of lumbar vertebral vBMD in the severity and advancement of LEPLs among common Chinese community. As the conjunct structure, the endplate is vital for maintaining the integrity of the vertebral trabeculae and disc. Damage to the endplate may impair not only the adjacent disc but the vertebra. Fujiwara et al. [5] have shown that LEPLs are associated with vertebral fractures and the healing of osteoporotic vertebral fractures. We find that the relationship between lumbar vBMD and LEPLs vanished after adjustment for covariates, suggesting that changes in BMD are not a protective or risk factor for LEPLs both in women and men. This is consistent with the result of Gungor that there was no significant relationship between the total number of Schmorl’s nodes per patient and the mean vBMD (*p* = 0.156) [10], and different from the result of Okano that higher TEPS (score 10 − 12, b = 14.2, *p* < 0.001) was shown to be independent contributors to vBMD [11]. The age difference demographic variance maybe accounts for this non-conformity.

In 2018, two common and certified nomenclature systems for endplate lesions had been almost recommended simultaneously by Brayda-Bruno [4] and Feng [8], respectively. Although Wang’s team contributed to the lumbar spine study both in vivo and cadaveric spines for many years [19, 24], Feng et. al considers that the different types of endplate defects differed in morphology, distribution patterns, and strength of association with disc degeneration, suggesting they represent different pathologies [8]. Nevertheless, the classification systems of Brayda-Bruno suppose that same as Pfirrmann’s classification of LDD, lesion grade from lower to higher is a progressive or continuous process. And our results using Brayda-Bruno’s classification also show that people with higher grades of lesions are older both men and women. Although the etiology of LEPLs is vague, our study is in line with the known theory that the majority of endplate lesions are asymptomatic, and is frequently incidental by radiological, and can partly suggest that the occurrence and development of LEPLs is a cumulative process. And the monistic study of the disease may reduce unnecessary controversy and confusion.

Although we used the more accurate strategy, QCT to measure vBMD in a large cohort of 750 healthy subjects, there are several limitations to this study. First, we executed a cross-sectional study,　whereas endplate and disc disorders and decreasing BMD being a continuing occurrence needs a longitudinal follow-up study to elaborate further on their relationship, which was not done in this study. Second, our study population was drawn from community-dwelling adults in the city of Beijing and might underestimate the prevalence of LEPLs in the general population, because LEPLs are associated with physical activity and economic well-being. Third, the loss of older people would likely have reduced the chance of showing significant relationships between evaluations of LEPLs and other variates.

In summary, LEPLs are also the common findings on lumbar MRIs in general population, particularly in men. The present results suggest that the presence and advance of LEPLs could be mainly attributed to LDH and male hipline, suggesting that treatment of LDH and avoidance of inappropriate physical activity may delay the progression of LEPLs. However, further studies are needed to evaluate the clinical implication of endplate lesions and remission of the back pain it causes.

## Data Availability

The data that support the findings of this study are available from the corresponding author but restrictions apply to the availability of these data, which were used under license for the current study, and so are not publicly available. Data are however available from the authors upon reasonable request and with permission of Beijing Jishuitan Hospital.

## Abbreviations

EPs: Endplates
LEPLs: Lumbar vertebral endplates lesions
LBP: Low back pain
LDH: Lumbar disc herniation
LDD: Lumbar disc degeneration
vBMD: Volumetric bone mineral density
QCT: Quantitative computed tomography
BMI: Body mass index
ANOVA: Analysis of variance
OLR: Ordinal logistic regression
